# Cyclodextrin and its derivatives as effective excipients for amorphous ulipristal acetate systems†

**DOI:** 10.1039/d1ra09420c

**Published:** 2022-03-23

**Authors:** Peng Wang, Yan Wang, Zili Suo, Yuanming Zhai, Hui Li

**Affiliations:** College of Chemical Engineering, Sichuan University Chengdu Sichuan China lihuilab@sina.com +86 028 85401207 +86 028 85405149; Sichuan Center for Disease Control and Prevention Chengdu Sichuan China; Analytical & Testing Center, Sichuan University P. R. China

## Abstract

Many efforts have been devoted to screening new solid-state forms of poorly soluble drugs in the pharmaceutical industry, thus modulating the drug properties without changing the pharmacological nature. It is a wise strategy to prepare amorphous series with cyclodextrin (CD) and its derivatives as excipients to enhance the aqueous solubility, dissolution, and bioavailability of water-insoluble drugs. In this study, four binary amorphous mixtures of ulipristal acetate (UPA) with CDs (β-CD, γ-CD, dimethyl-β-CD, hydroxypropyl-β-CD) were prepared by the co-milling method and characterized in the solid-state. According to powder X-ray diffraction (PXRD) and differential scanning calorimetry (DSC), UPA existed in the noncrystalline form in the four binary amorphous mixtures. Fourier transform infrared spectroscopy (FT-IR) and nuclear magnetic resonance (NMR) indicated that UPA interacted with the four CDs, which was also verified by molecular docking. Compared with the crystalline and amorphous UPA, the solubility, dissolution, and stability of the drug in the four amorphous UPA systems were significantly improved, so they were considered potentially advantageous solid forms. Our research shows that CDs can be used as new effective excipients in amorphous systems for active pharmaceutical ingredients (API).

## Abstract 2

Probing into the amorphous mixtures of ulipristal acetate and cyclodextrins.

## Graphical abstract

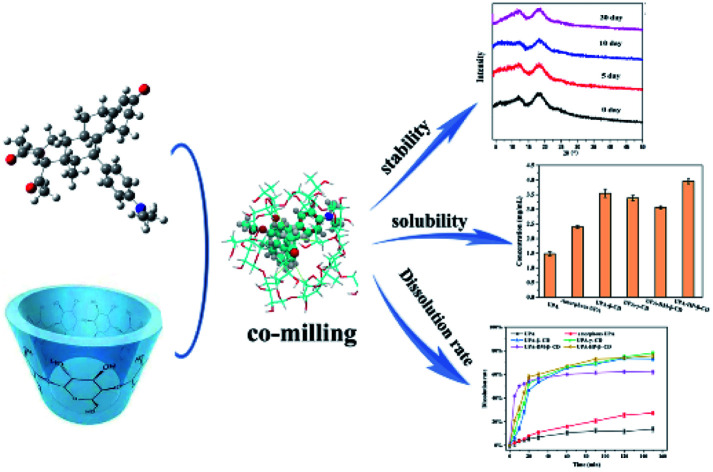

## 1. Introduction

Approximately 90% of current candidates exhibit limited water solubility, which often causes oral absorption problems and increases the side effects of poorly water-soluble drugs.^1^ Converting crystalline drugs into an amorphous form is an effective solubilization technology.^2^ In the amorphous state, the absence of long-range molecular order and the higher Gibbs free energy in amorphous drugs are the main reason for their higher solubility, and hence dissolution rate and bioavailability.^3^ However, due to the high thermodynamic energy, most amorphous drugs alone are not sufficiently physically stable during the process, storage, and dissolution.^4^

In some cases, polymers with a high glass transition temperature are often added to prepare amorphous solid dispersions (ASDs) in order to prevent the crystallization of an amorphous drug.^5^ However, this technique has some disadvantages that cannot be ignored.^6^ For example, the limited miscibility of the drug with the polymer sometimes requires the addition of a large amount of polymer, which is disadvantageous for the usability, manufacturing, and cost.^7^ Besides, ASDs also have problems of poor long-term stability as well as difficulty in manufacturing and processing.^8^ Co-amorphous systems have been extensively studied in the pharmaceutical industry because of their excellent properties, such as promoting drug solubilization, enhancing drug stability, and improving drug delivery and absorption.^9,10^ They are combinations of a drug and other small molecules, such as organic acids, amino acids, and other drugs.^11,12^ A drug and small molecule may interact *via* hydrogen bonds or other noncovalent bonds, which can reduce the mobility of long-range disordered molecules in amorphous drugs, thereby improving their physical properties.^13^ Whilst there are clear advantages of co-amorphous systems, there are also some challenges, such as the high hygroscopicity of small molecules that sometimes affect their stability, thus limiting their practical applications.^14^

Cyclodextrins (CDs) have been recognized as pharmaceutical excipients, which are cyclic oligosaccharides composed of d-glucopyranose units with a slightly conical hollow cylinder stereoscopic ring structure.^15,16^ In terms of the number of d-glucose units, the common natural CD can be classified as alpha-CD (α-CD), beta-CD (β-CD), and gamma-CD (γ-CD).^17^ With the development of pharmaceutics, more CD derivatives have been developed and applied. Among those derivatives, 2,6-dimethyl-β-CD (DM-β-CD) and 2-hydroxypropyl-β-CD (HP-β-CD) with good water solubility are the most commonly used.^18^ They all have satisfactory inclusion ability, surface activity, and cavity depth suitable for drug loading.^19^ Due to the special molecular structure with hydrophilic exteriors and hydrophobic internal cavity,^20^ CDs exhibit vast application potential in the pharmaceuticals, drug delivery systems, and chemical industries.^21,22^ The high density of hydroxyl groups makes it possible to interact with drugs groups, which also makes it a versatile molecule for modifying drug properties.^23^ CDs can effectively increase the solubility and dissolution rate in an aqueous medium of compounds with low solubility and pharmacodynamic properties of drug molecules.^24^ More generally, the importance of substitution patterns may also affect the structure–activity relationships on poorly soluble drugs (interactions, complexations, toxicity, stability, and solubilizing effects…).^25^ However, most of the current research focuses on the applications of CDs, and there are relatively few studies on the screening and properties of binary amorphous materials prepared by CDs.

Ulipristal acetate (UPA) is the first progestin receptor modulator after unprotected sexual intercourse within 5 days. It has a high inhibitory effect on progesterone receptors.^26^ UPA belongs to BCS II class compound and most of the commercially available raw materials are crystalline, whose low solubility limits its clinical application.^27^ As a contraceptive, it is important to improve the dissolution and release of UPA. Due to the small cavity structure, α-CD is not suitable for the system of larger molecules like UPA. Therefore, β-CD, γ-CD, DM-β-CD, and HP-β-CD were used as excipients in this paper, we have prepared four UPA–CD binary amorphous materials by the co-milling method. Powder X-ray diffraction (PXRD) was used to monitor the preparation process while differential scanning calorimetry (DSC), infrared spectroscopy (FT-IR), and nuclear magnetic resonance (NMR) were used to characterize. Besides, the interaction between UPA and CDs was investigated by molecular docking. The properties of the different solid drug forms were investigated from three aspects of solubility, dissolution rate, and stability to evaluate their potential as a drug product with high bioavailability.

## 2. Experimental

### 2.1. Materials

**Fig. 1:**
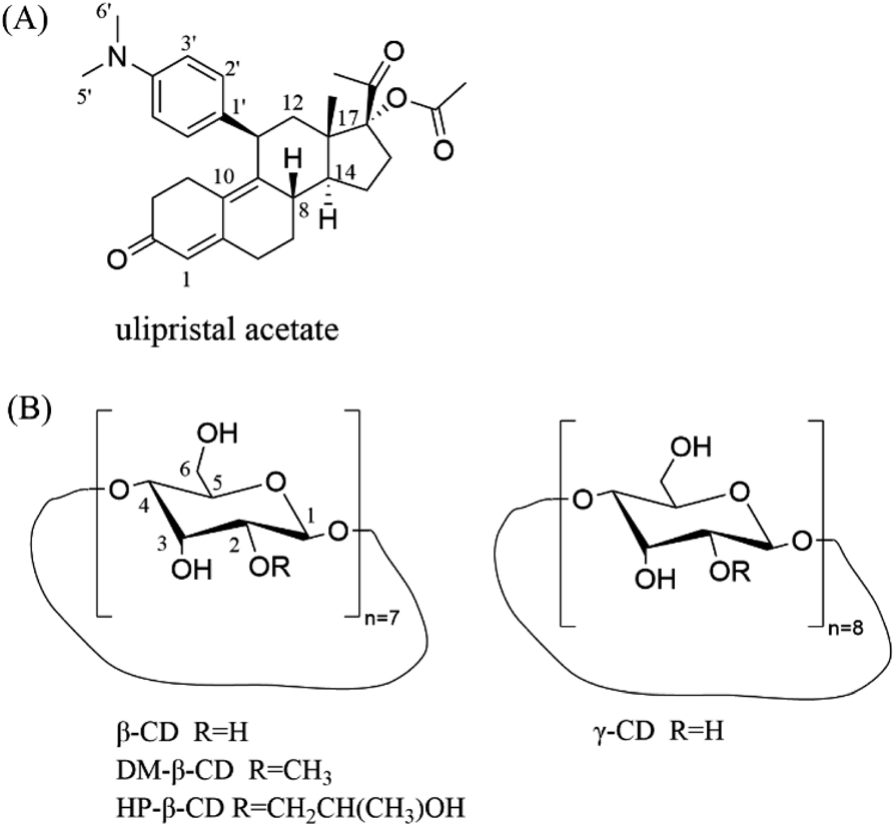
Chemical structures of (A) UPA and (B) CDs.

The main drug crystalline UPA (purity ≥99%) was obtained from Chengdu D-innovation Pharmaceutical Technology Co., Ltd. β-CD, γ-CD, DM-β-CD, and HP-β-CD was purchased from Kelong Company, Ltd (Chengdu, China) (Fig. 1). Phosphoric acid and potassium dihydrogen phosphate (chromatographic pure) were purchased from Ourchem (Chengdu, China) and Fisher Scientific (Fair Lawn, NJ, USA), respectively. Water used in the experiment was purified using a Milli Q water purification system (Millipore Corp., MA, USA).

### 2.2. Methods

#### 2.2.1 Preparation of UPA solid formulations

Amorphous UPA solid formulations were prepared by neat milling (NM) in a ball mill (LNMN-QM 0.4 L, Heishan Xinlitun Agate Handicrafts Co., Ltd, China). The rotation speed was set to 500 rpm. Alternating milling time (10 min) and pause time (2 min) was applied to prevent local overheating of the solid samples during the mechanical lapping. About 1 g UPA was weighed in agate balls jars (50 cm^3^). The crystalline UPA was ground until the diffraction peak disappears completely, thus the amorphous UPA was obtained. Subsequently, a total mass of 1 g of the UPA and four CDs at molar ratios of 1 : 1 were placed into agate ball jars and co-milling without solvent added. All the solid powders were withdrawn and analyzed by PXRD. The grinding stopped when all diffraction peaks disappeared. Physical mixtures (PM) were prepared by geometric mixing of crystalline UPA with each CD at equal mass ratios using a motor and pestle for a few minutes.

#### 2.2.2 Physical stability studies

The stability of amorphous UPA and four UPA–CD amorphous mixtures at temperature (60 °C), humidity (40 °C, 75% RH), and photolysis (25 °C, 1 200 000 lux h) were investigated. The stability of different solid forms was monitored by PXRD at 5, 10, and 30 days under different conditions. At the same time, the impurities in UPA during the different storage states were quantitatively analyzed, and the relevant substances of different UPA solid forms were also detected by the high-performance liquid chromatography (HPLC).

#### 2.2.3 Solubility studies

The excess samples were added to 10 mL artificial gastric juice (pH = 2) in Erlenmeyer flasks, thus preparing the supersaturated sample solutions. The supersaturated solutions were stirred at 100 rpm for 48 h at a 37 °C water bath, filtered through a 0.45 μm syringe, and diluted appropriately. The solubility of UPA in different solid forms was determined by HPLC according to the standard curve.

#### 2.2.4 Dissolution studies

*In vitro* dissolution rates of different UPA solid forms were measured by a ZRC-8D dissolution tester (Chuangxing, Tianjin, China). Amorphous UPA (30 mg) and UPA–CD combinations with equivalent measurement of UPA were accurately weighed into a 900 mL dissolution medium (artificial gastric juice with pH = 2). At 5, 10, 15, 20, 30, 60, 90, 120, and 150 min at the beginning of the experiment, 2 mL solution was taken out with a syringe and filtered through a 0.45 μm syringe. At the same time added 2 mL artificial gastric juice. At least three parallel experiments were performed for each measurement. The cumulative dissolution rate can be calculated by the following formula:1
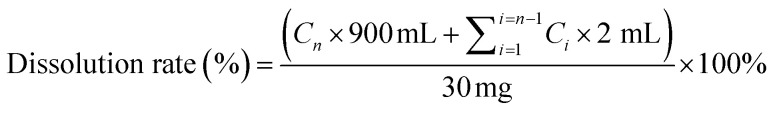


### 2.3 Analytical techniques

#### 2.3.1 Powder X-ray diffractometry (PXRD)

PXRD measures were performed to monitor for amorphous formation of the UPA solid form samples by using a diffractometer (X'Pert PRO; PANalytical, Almelo, Netherlands) with a PIXcel one-dimensional detector and Cu Kα radiation. The acceleration voltage was 40 kV and the tube current was 40 mA. Diffraction data were collected in a 2*θ* range of 4°–50° with a step size of 0.026°.

#### 2.3.2 Scanning electron microscopy (SEM)

The morphological of the different UPA solid form samples were obtained under the SEM (JSM-7500F; JEOL, Tokyo, Japan) at 15.0 kV. Electrically conductive samples were prepared by coating with a thin gold layer in a vacuum before the examination.

#### 2.3.3 Thermal analysis

Thermogravimetric analysis (TGA) was obtained on a TG209F1 instrument (Iris; Netzsch, Selb, Germany). Samples (5–8 mg) were measured from 40 °C to 600 °C using an analyzer in an aluminum crucible at a constant rate of 10 °C min^−1^ under N_2_ purge (60 mL min^−1^). DSC thermograms were measured by differential scanning calorimeter Q200 (TA Instruments Co., New Castle, DE, USA). After grinding, the dry samples (2–4 mg) were accurately weighed and placed in an aluminum crucible, the crucible cover was covered and the samples were prepared with a confidential seal. Under N_2_ purging (flow rate of 50 mL min^−1^), the samples were heated from 40 °C to 220 °C at a rate of 10 °C min^−1^, and the DSC curves were recorded.

#### 2.3.4 Fourier transform infrared spectroscopy (FT-IR)

FT-IR spectra were obtained using a Nicolet 6700 FT-IR spectrometer (Thermo Scientific, WI, USA). The dried UPA solid form samples were dispersed in KBr, ground evenly in mortar and pestle and the powder size was fine enough, and the disk was prepared by applying a pressure of about 1000 psig. A total of 64 scans were performed over a range of 4000–400 cm^−1^ with a spectral resolution of 4 cm^−1^.

#### 2.3.5 Nuclear magnetic resonance (NMR)

^1^H NMR spectra of the samples were obtained at 295 K with a Bruker Avance 400 spectrometer (Germany) at 400 MHz. DMSO-d_6_ was used to dissolve all the samples.

#### 2.3.6 HPLC analysis

The test conditions for quantitative analysis of the UPA were determined by external calibration at 40 °C using an HPLC Agilent 1260 instrument (Agilent Technologies, Inc., USA) equipped with an Xtimate® C18 column (250 mm × 4.6 mm, 5 μm), and an acetonitrile/water (90 : 10) mixture as mobile phase 1 mL min^−1^. The retention time is 7.59 min.

The related substances of different UPA solid form samples during storage were quantified by external calibration at 40 °C using an Xtimate® C18 column (250 mm × 4.6 mm, 5 μm). The mobile phase was a mixture of acetonitrile and water (70 : 30 v/v) at a flow rate of 1.0 mL min^−1^ and the retention time is 5.25 min (Fig. S1†).

## 3. Results and discussion

### 3.1. Characterization of solid dispersion

#### 3.1.1 PXRD

Different amorphous UPA systems were prepared by mechanochemical methods. As shown in Fig. S2,† the characteristic diffraction peaks of the crystalline UPA gradually weaken as the milling time increases, indicating that the crystalline UPA gradually changed to an amorphous state. After grinding for 180 min, the diffraction peaks of UPA were not detected and two broad diffusion amorphous halos were observed, indicating the formation of amorphous UPA.

**Fig. 2:**
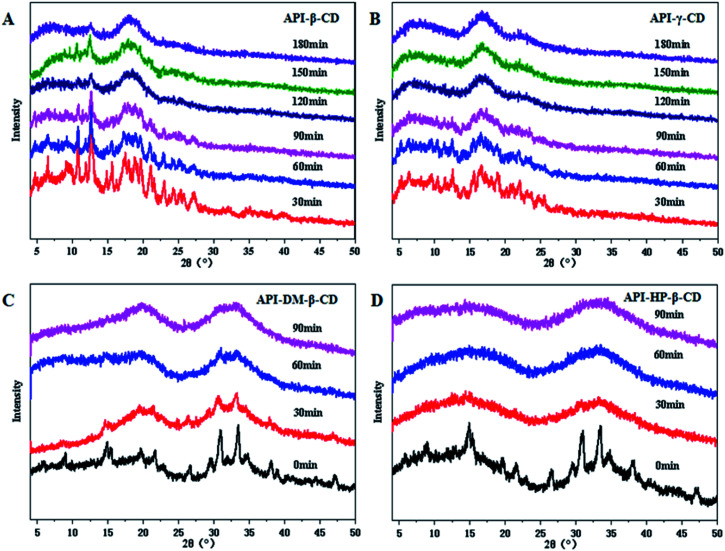
Preparation of amorphous systems of PXRD patterns of UPA-β-CD (A), UPA-γ-CD (B), UPA-DM-β-CD (C), and UPA-HP-β-CD (D) obtained at different time points during milling.

Four CDs and their derivatives (β-CD, γ-CD, DM-β-CD, HP-β-CD) were selected to evaluate whether they could successfully convert into the binary amorphous systems. The co-milled solid powders were monitored by PXRD to confirm the successful preparation of the amorphous mixtures. As illustrated in Fig. 2, the four UPA–CD systems were gradually transformed into amorphous counterparts as the milling time increased. The formation time of UPA-γ-CD, UPA-DM-β-CD, and UPA-HP-β-CD amorphous mixed systems was greatly shortened. After grinding UPA-β-CD for 2 hours, the diffraction peaks also disappeared, which is attributed to dilution and surface coverage of the UPA particles by CDs.^28^ Moreover, UPA is presented in the amorphous form in the four UPA–CD binary amorphous systems. The phenomenon initially indicated the formations of amorphous UPA and UPA-CD amorphous mixtures (CM). UPA interacts with CDs to a certain extent, which promotes the formation of amorphous systems.

#### 3.1.2. Morphology analysis

**Fig. 3:**
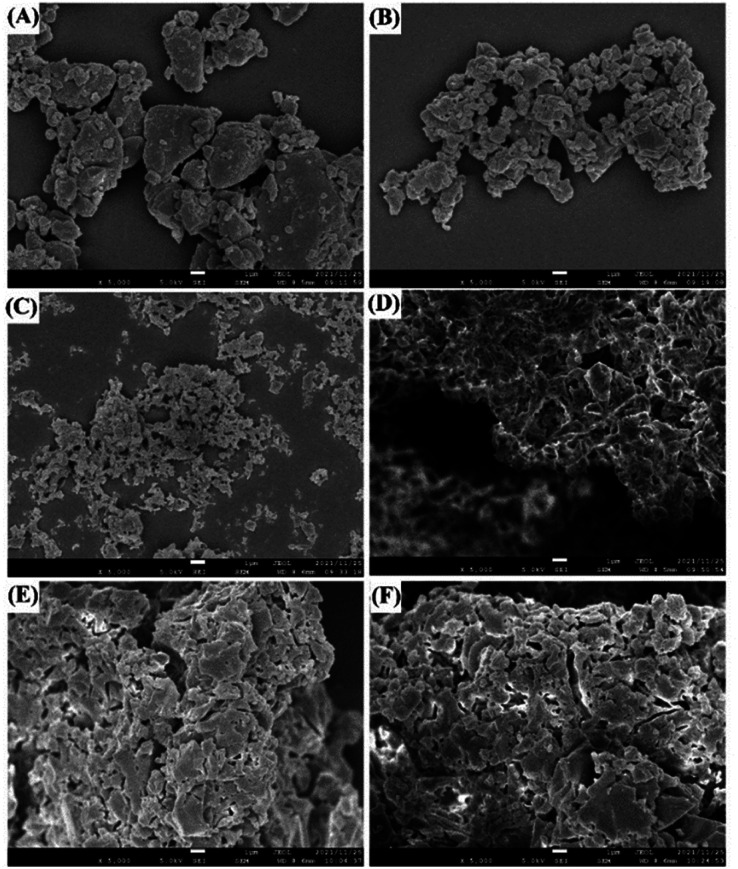
SEM photographs of UPA (A), amorphous UPA (B), UPA-β-CD amorphous (C), UPA-γ-CD amorphous (D), UPA-DM-β-CD amorphous (E), and UPA-HP-β-CD amorphous (F).

The micromorphological characteristics of the different UPA solid samples were observed through SEM. The amorphous morphologies of UPA and four UPA–CD systems were shown in Fig. 3. It is observed that the crystalline UPA presents a regular block structure. However, as shown in Fig. 3B, after grinding, the particle size of the amorphous UPA becomes smaller and the particles gather together, presenting an irregular block particle structure.

There are significant differences in the structures of several single components. In our previous study, it was observed that β-CD was an irregular massive crystal, while HP-β-CD was a granular sphere with the cavity, DM-β-CD was spherical and accompanied by a large number of spherical ruptures, and HP-β-CD was a hollow spherical structure.^29^ However, the amorphous samples co-milled with UPA and CDs showed irregular block structures with different sizes and uneven surfaces, which were completely different from the morphology of single UPA and four CDs. Amorphous UPA was agglomerated together due to the static electricity produced during the grinding process. The amorphous mixture of UPA and four CDs have a uniform size, which is dispersed evenly. This finding may be due to the introduction of CD increasing the hydrophilicity of the samples, thus reducing static electricity and making the grinding more uniform.^30^

#### 3.1.3. Thermal analysis

**Fig. 4:**
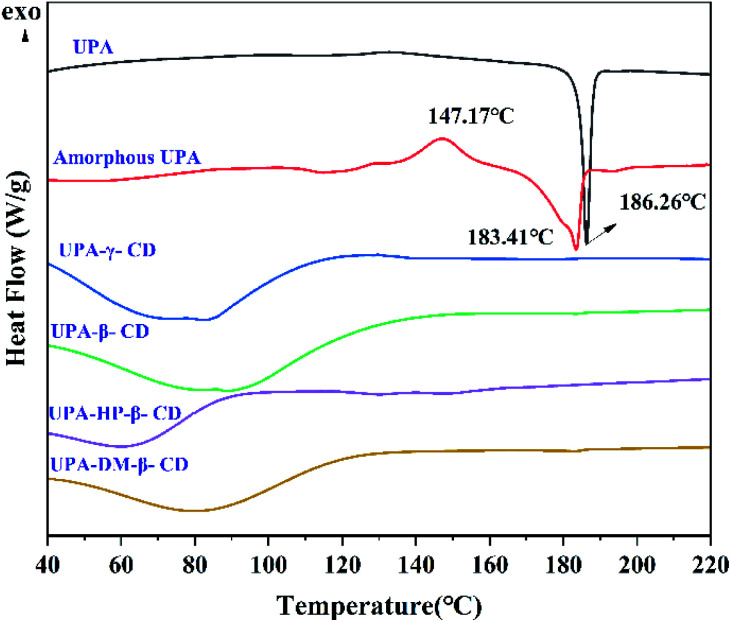
DSC curves of pure UPA, amorphous UPA, the UPA–CD amorphous.

TGA and DSC were used to analyze the thermal properties of UPA in the different solid-state, and the information of the change and molecular structure during the heating process could be obtained. As shown in Fig. S3,† when the temperature was higher than 280 °C, the quality of crystalline UPA samples decreased rapidly, which was caused by the rapid decomposition of UPA molecules and partial volatilization of decomposition products. The TGA curves of the four UPA–CD systems continued downward from 150 °C until the sample mass stabilized at 500 °C, with a mass loss of nearly 100%. This was because of the vaporization of UPA and the simultaneously thermal decomposition of CDs. Fig. 4 showed the DSC curves of UPA, CDs, UPA–CDs binary amorphous mixtures. Crystalline UPA only displayed an endothermic melting peak at 186.26 °C, indicating that the bulk drug had no solvent residue and its melting point was 186.26 °C. Amorphous UPA underwent a vitrification transformation process at about 104.65 °C and recrystallized at 147.17 °C, which transformed into UPA crystal form and then melted at 183.41 °C, corresponding to the melting point peak of UPA. Four UPA–CD binary amorphous systems had no melting point peak of UPA, which further confirmed the amorphous structure of the binary system prepared by PXRD.

### 3.2. Interactions between UPA and CDs

#### 3.2.1 FT-IR analysis

**Fig. 5:**
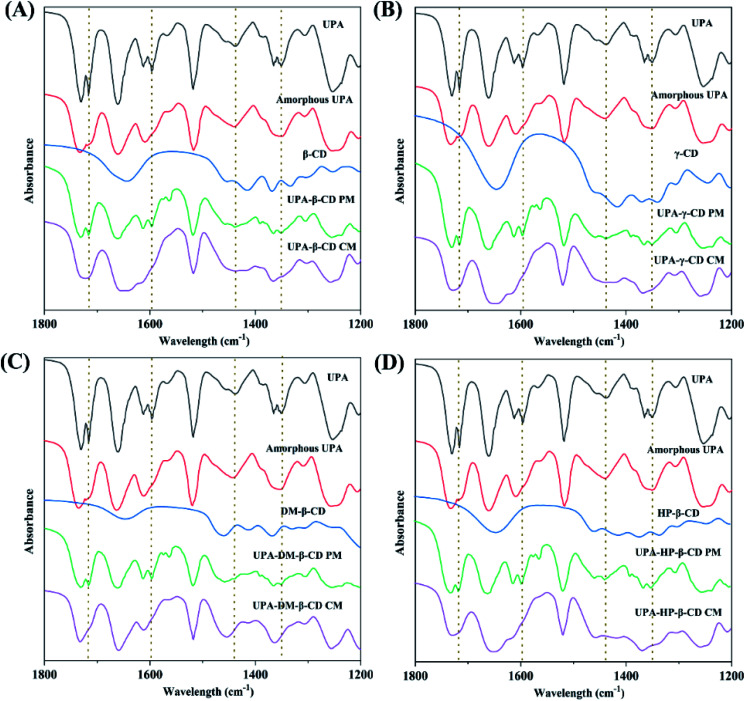
FT-IR spectra of UPA-β-CD (A), UPA-γ-CD (B), UPA-DM-β-CD (C), and UPA-HP-β-CD (D). Each picture from top to bottom: crystalline UPA, amorphous UPA, CD, UPA-CD PM, and UPA-CD CM.

Co-milling may lead to changes in their oscillating dipoles between different components, which will be represented by changes in the frequency of the interacting groups in the FT-IR spectrum. After UPA formed amorphous complexes with CDs, the characteristic peaks of UPA may decrease, shift, or disappear. The FT-IR spectra of UPA, β-CD, γ-CD, HP-β-CD, DM-β-CD, their physical mixtures, and their amorphous complexes are shown in Fig. 5. UPA showed strong stretching vibration peaks at two wavenumbers of 1730 cm^−1^ and 1660 cm^−1^, which were two characteristic absorptions of C=O in UPA respectively. The C=O peak intensity of amorphous UPA decreased significantly. The FT-IR spectra of the physical mixtures corresponded to the superposition of the FT-IR spectra of pure UPA and CDs. The FT-IR spectra of UPA-β-CD, UPA-γ-CD, UPA-DM-β-CD, and UPA-HP-β-CD showed some characteristic absorption peaks of UPA and CD, but the intensity of the UPA peak decreased significantly. Compared to the corresponding physical mixtures, after forming the UPA-β-CD amorphous, the absorption peaks of UPA at 1716 cm^−1^ for strong C=O stretching vibration band, at 1598 cm^−1^ for C–O bending vibration, and at 1351 cm^−1^ for N–C covalent stretching band were masked by β-CD. The absorption peaks of UPA at 1438 cm^−1^ for C–H asymmetrical bending vibration and at 1365 cm^−1^ for C–H symmetric bending vibration showed reduced intensity and merged into a peak at 1456 cm^−1^. The same results were obtained in the FT-IR spectra of UPA-γ-CD, UPA-DM-β-CD, and UPA-HP-β-CD amorphous systems. The results showed that the interaction between UPA and CDs had a certain influence on the stretching vibration of the group, and then changed the peak shape or peak position. These results indicated the formation of binary amorphous systems.

#### 3.2.2 ^1^H NMR analysis

^1^H NMR chemical shift of UPA–CD complexes may affect by the interaction of UPA and CDs. The ^1^H NMR spectra of UPA, HP-β-CD and UPA-HP-β-CD amorphous complexes are shown in Fig. S5.† It can be found that all the UPA protons are shown in the spectrum of the UPA–CD complexes, which confirms the presence of UPA in the amorphous complexes. Table 1 shows the chemical shifts and their changes in the complexes. In the presence of UPA, the ^1^H NMR spectra of CDs exhibit an upfield shift of their H-3 and H-5 protons. The obvious changes of the CD internal protons indicate that the UPA molecule may have embedded into the CD cavities. The benzene and methyl protons of UPA protons also exhibited slight chemical shift changes in presence of the CDs and suggested that some protons of UPA were preferably involved in the interaction with CDs. The ^1^H NMR results indicated that part of the UPA was possibly accommodated into the CD cavity to form the amorphous systems.

**Table 1:** ^1^H NMR chemical shifts (*δ*, ppm) and Δ*δ* values of CDs and UPA protons in single state and in complexes

Samples		UPA				CD	
		H-2′	H-3′	H-1	H-5′, H-6′	H-3	H-5
UPA-β-CD	Free	6.765	6.419	5.452	3.122	3.629	3.544
	Complex	6.777	6.412	5.474	3.143	3.408	3.367
	Δ*δ*	0.012	−0.007	0.022	0.021	−0.221	−0.177
UPA-γ-CD	Free	6.765	6.419	5.453	3.122	3.607	3.564
	Complex	6.766	6.417	5.450	3.133	3.521	3.385
	Δ*δ*	0.001	−0.002	−0.003	0.011	−0.086	−0.179
UPA-HP-β-CD	Free	6.765	6.419	5.452	3.122	3.557	3.493
	Complex	6.768	6.418	5.455	3.117	3.485	3.334
	Δ*δ*	0.003	−0.001	0.003	−0.005	−0.072	−0.159
UPA-DM-β-CD	Free	6.765	6.419	5.452	3.122	3.622	3.519
	Complex	6.767	6.418	5.454	3.136	3.399	3.319
	Δ*δ*	0.002	−0.001	0.002	0.014	−0.223	−0.200

#### 3.2.3 Molecular docking

After molecular docking in YASARA cluster analysis, the docking results of UPA with β-CD, γ-CD, DM-β-CD, and UPA-HP-β-CD showed a variety of possible conformations. According to the order of scoring, the first conformation is regarded as the best conformation for further analysis. The binding energy of the best conformation between the drug and the CDs is 6.39 kcal mol^−1^ for UPA-β-CD, 6.27 kcal mol^−1^ for UPA-γ-CD, 5.72 kcal mol^−1^ for UPA-DM-β-CD, and UPA-HP-β-CD is 6.83 kcal mol^−1^. Due to the similar structure of β-CD and γ-CD, the interaction energy of the two complexes is close, and the slight difference comes from the difference in cavity size. The binding energy of UPA-DM-β-CD and UPA-HP-β-CD was higher due to the difference of substituent modification. The highest binding energy between UPA and HP-β-CD indicates that among the four CDs, UPA and HP-β-CD have the strongest interaction. Thus, UPA can be better dispersed in HP-β-CD and the drug crystallization can be better inhibited.

**Fig. 6:**
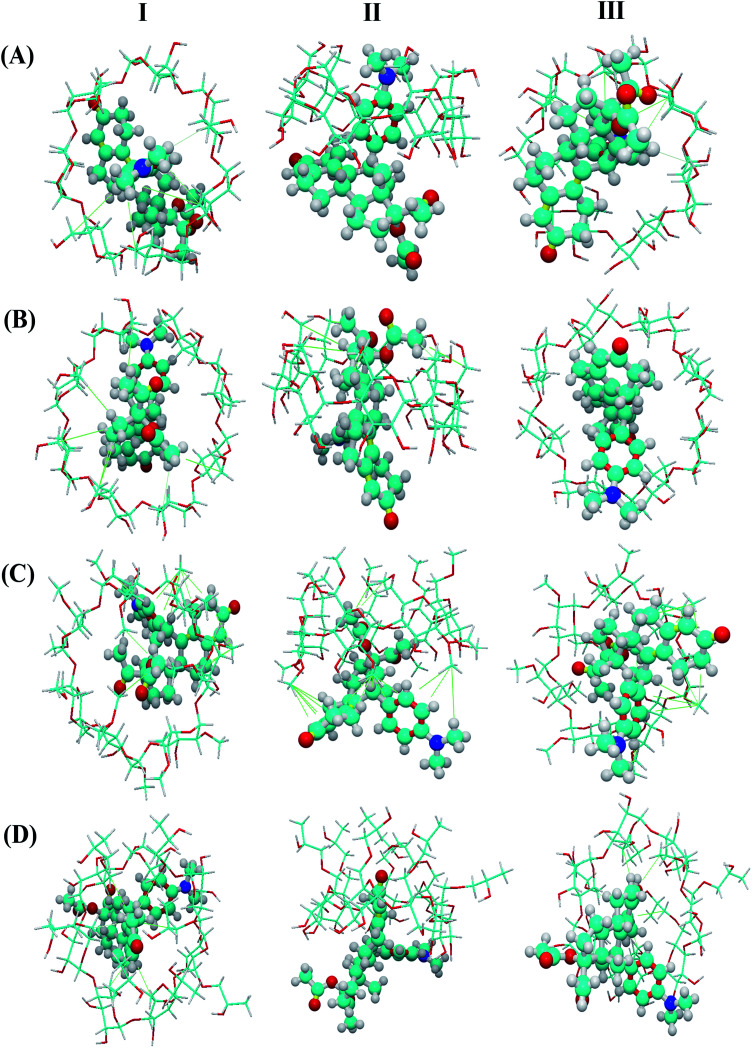
Molecular models of the inclusion complexes of UPA with β-CD, γ-CD, DM-β-CD, and UPA-HP-β-CD (I, II and III were corresponding to the top, side and bottom views of the models).

As shown in Fig. 6, host–guest complexation can be formed but the depth of inclusion of the guest molecule into the CD cavity varies. The results showed that β-CD and γ-CD entered the cavity more deeply than DM-β-CD and HP-β-CD, which may be related to the molecular size of CD. Besides, the UPA molecules and CD molecules (β-CD, γ-CD, DM-β-CD, and HP-β-CD) adjust the conformation each other and combine to form a stable complex under the action of hydrophobic force. These results theoretically confirmed that there was a certain degree of interaction between UPA and the four CDs.

### 3.3. Physical stability

This study explored the effects of temperature, humidity, and photolysis on the different UPA amorphous solid forms. The stabilities of the different amorphous samples were monitored and the results were shown in Fig. S6–S10.† After 10 days of storage at temperature and humidity, the pure amorphous UPA showed a tendency of crystallization, which is confirmed by the change of PXRD diffraction peaks. Amorphous UPA maintained an amorphous halo under light storage conditions, indicating that light had little effect on the stability of amorphous UPA, and temperature and humidity were the main reasons for their stability. The four UPA–CD amorphous mixture systems showed excellent stability at different stress conditions. Under the conditions of temperature and humidity for 30 days, four UPA–CD systems maintained an amorphous halo and no obvious diffraction peak appeared. The improved stability is due to the hollow structure of CDs, which is the result of wrapping UPA in the process of forming an amorphous mixture, thus having a certain protective effect.

Table 2 showed the contents of related substances in different UPA amorphous systems under temperature, humidity, and photolysis. According to the data, the total impurity contents of amorphous UPA are 1.00%, 0.96%, and 2.76%, respectively, after being stored under temperature, humidity, and photolysis for 10 days, which is slightly higher than that of crystalline UPA. The single impurity of UPA was *N*-desmethylulipristal acetate with a run time of 5.6 min (Fig. S1†). The total impurity and single impurity content of the four amorphous UPA–CD systems were lower than that of the crystalline UPA under photolysis and close to that of the crystalline UPA under temperature and humidity conditions, indicating that the addition of CDs did not change the properties of the UPA, and the growth rate of related substances was not higher than that of the UPA. The stabilizing effect of CDs on amorphous drugs may due to the interaction of CDs with UPA, thereby interfering with the interaction between drug molecules and enhancing the stability of systems.

**Table 2:** Stability results of influencing factors of UPA and amorphous compounds

Sample		Initial	Temperature 60 °C	Humidity 75% RH	Photolysis 1 200 000 lux h
UPA	Single impurity%	0.53	0.70	0.69	1.62
	Total impurity%	0.67	0.94	0.94	2.76
Amorphous UPA	Single impurity%	0.55	0.74	0.70	1.70
	Total impurity%	0.69	1.00	0.96	2.94
UPA-β-CD	Single impurity%	0.54	0.74	0.68	1.46
	Total impurity%	0.68	1.00	0.93	2.45
UPA-γ-CD	Single impurity%	0.55	0.72	0.71	1.40
	Total impurity%	0.69	1.03	0.96	2.34
UPA-DM-β-CD	Single impurity%	0.54	0.73	0.68	1.40
	Total impurity%	0.68	1.00	1.13	2.33
UPA-HP-β-CD	Single impurity%	0.56	0.74	0.68	1.32
	Total impurity%	0.72	1.00	0.94	2.21

### 3.4 Solubility and dissolution tests

**Fig. 7:**
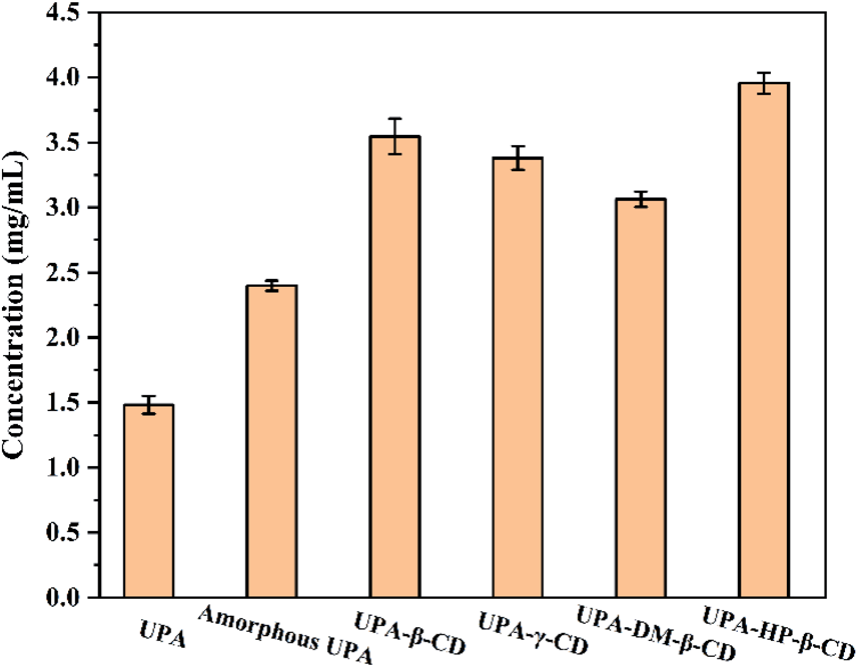
Solubility histograms of crystalline UPA, amorphous UPA, and UPA-CD in artificial gastric juice at pH = 2 (mean ± SD, *n* = 3).

Improving the water solubility of drugs is one of the main reasons for preparing new solid forms of UPA. The apparent solubility results are shown in Fig. 7. Compared with crystalline UPA, amorphous UPA and UPA–CD binary systems displayed different degrees of advantage in solubility. The solubility of amorphous UPA was significantly improved compared with 1.48 mg mL^−1^ of crystalline UPA in simulated gastric juice, which was 1.62 times higher than those of the UPA.

DM-β-CD was the fastest among them, 40% of the drug was released within 5 min, and the cumulative dissolution rate of UPA-DM-β-CD was 62% after 150 min, which represented a 4.43-fold and 1.63-fold increase compared with crystalline and amorphous UPA, respectively. The cumulative dissolution rate of the UPA-HP-β-CD binary amorphous system was similar to that of UPA-β-CD, and UPA-γ-CD amorphous, both about 75%, which was 5 times that of UPA, indicating that the interaction between UPA and CD molecules greatly promoted the dissolution and release of UPA. Among the four UPA–CD amorphous mixtures, UPA-HP-β-CD showed the best solubility advantages and higher cumulative dissolutions over pure crystalline drugs.

The UPA-HP-β-CD amorphous preparation showed a significant advantage, which was 2.8 times (3.95 mg mL^−1^) higher than the crystalline UPA. The interaction between UPA and CDs promoted the dissolution of UPA and improved the solubility of API. The solubility order of the samples was UPA-HP-β-CD > UPA-β-CD > UPA-γ-CD > UPA-DM-β-CD > amorphous UPA > crystalline UPA. These results indicated that the solubility of the binary amorphous system was not only attributed to the amorphous structure of UPA, but also to the addition of CDs.

**Fig. 8:**
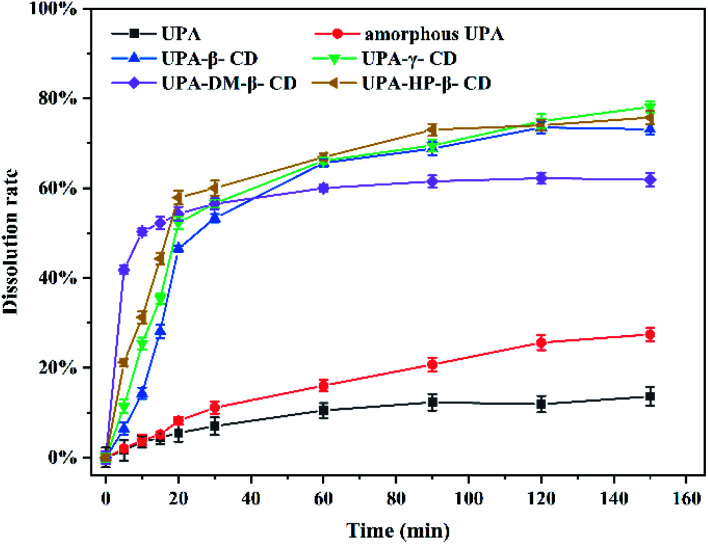
Dissolution rate curves of crystalline UPA, amorphous UPA, and UPA–CD amorphous in an artificial gastric juice at pH = 2.

Fig. 8 shows the dissolution rates of the different UPA amorphous samples. Crystalline UPA only exhibited a cumulative release of 14%. Compared with crystalline UPA, the cumulative dissolution of amorphous UPA is improved to a certain extent, which is about 38%. The dissolution rate of UPA-DM-β-CD was the fastest among them, 40% of the drug was released within 5 min, and the cumulative dissolution rate of UPA-DM-β-CD was 62% after 150 min, which represented a 4.43-fold and 1.63-fold increase compared with crystalline and amorphous UPA, respectively. The cumulative dissolution rate of the UPA-HP-β-CD binary amorphous system was similar to that of UPA-β-CD, and UPA-γ-CD amorphous, both about 75%, which was 5 times that of UPA, indicating that the interaction between UPA and CD molecules greatly promoted the dissolution and release of UPA. Among the four UPA–CD amorphous mixtures, UPA-HP-β-CD showed the best solubility advantages and higher cumulative dissolutions over pure crystalline drugs.

## 4. Conclusions

In this study, CDs and their derivatives were selected as effective excipients in poorly soluble drug UPA amorphous system. Four UPA–CD amorphous systems were successfully prepared by co-milling and characterized through PXRD, SEM, DSC, TGA, FT-IR, and ^1^H NMR. Our results revealed that the novel binary UPA–CD formulations were showed good performance compared with the crystalline and amorphous UPA. The low solubility of crystalline UPA and poor physical stability of amorphous UPA were effectively improved in the four UPA–CD binary amorphous mixture systems. The stability of UPA-β-CD, UPA-γ-CD, UPA-DM-β-CD, and UPA-HP-β-CD was better than that of amorphous UPA under temperature, humidity, and photolysis. The addition of CDs did not change the properties of the UPA, and the growth rate of related substances was not higher than that of UPA. Among the four amorphous mixtures, the UPA-HP-β-CD amorphous showed solubility advantages and it might be in favour of its oral bioavailability. The interaction between UPA and CDs greatly promoted the dissolution and release of UPA. These researches on the UPA–CDs amorphous systems presented a safe and effective formulation technology for the development of new drug solid forms, which is a promising formulation for improving oral absorption.

## Author contributions

Peng Wang: investigation, visualization, writing – original draft. Yan Wang: investigation, supervision. Zili Suo: investigation, supervision. Yuanming Zhai: writing – review and editing. Hui Li: writing – review and editing.

## Conflicts of interest

There are no conflicts to declare.

## Supplementary Material

RA-012-D1RA09420C-s001
