# Supplementary material for: Cyclodextrin and its derivatives as effective excipients for amorphous ulipristal acetate systems
Source: RSC Adv. 2022 Mar 23;12(15):9170–8. doi: 10.1039/d1ra09420c (PMC8985104; doi:10.1039/d1ra09420c)
Supplement: RA-012-D1RA09420C-s001 [file RA-012-D1RA09420C-s001.pdf]

Electronic Supplementary Material (ESI) for RSC Advances.
This journal is © The Royal Society of Chemistry 2022

Electronic Supplementary Information (ESI)

Cyclodextrin and its derivatives as effective excipients for the Ulipristal acetate amorphous systems

Peng Wang[1], Yan Wang[2], Zili Suo[1], Yuanming Zhai[3], and Hui Li[1]*

1.College of Chemical Engineering Sichuan University, Chengdu 610065, PR China

2. Sichuan Center for Disease Control and Prevention, Chengdu Sichuan, China.

3. Analytical & Testing Center, Sichuan University, P.R. China

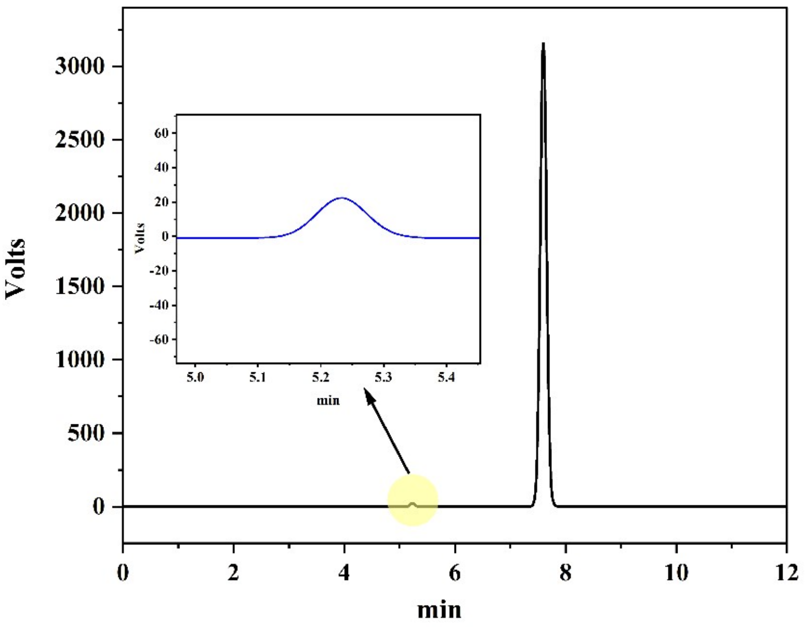

Fig. S1 HPLC spectrogram of the UPA under a certain concentration. Part of the enlarged image is the peak of N-desmethylulipristal acetate.

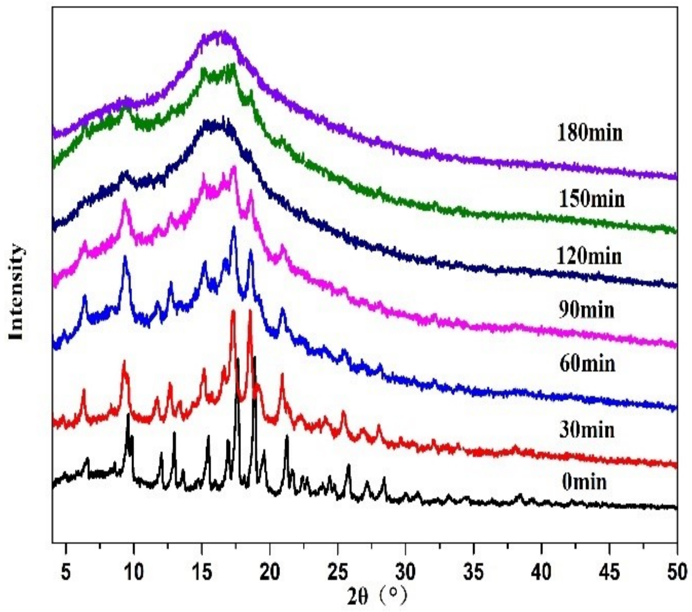

Fig. S2 Preparation of amorphous UPA

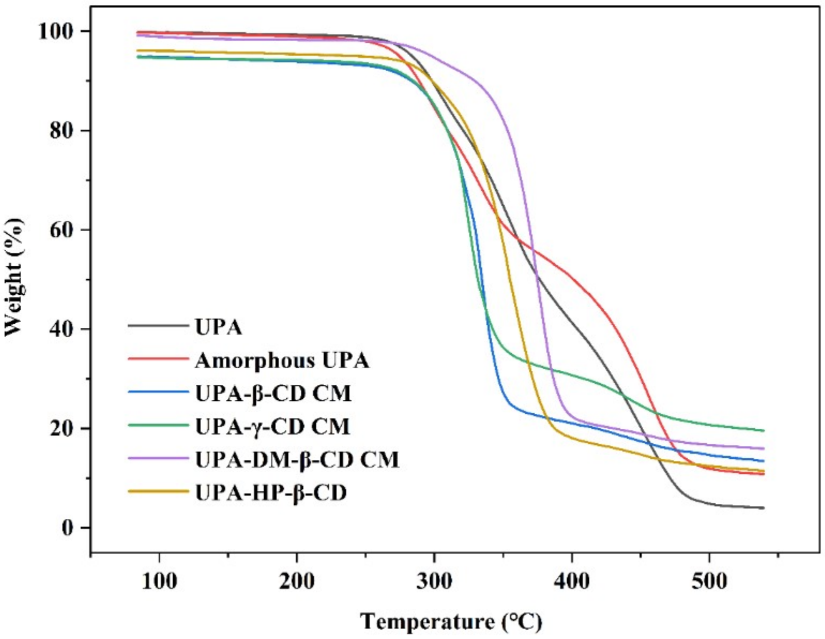

Fig. S3 TGA curves of different UPA solid forms.

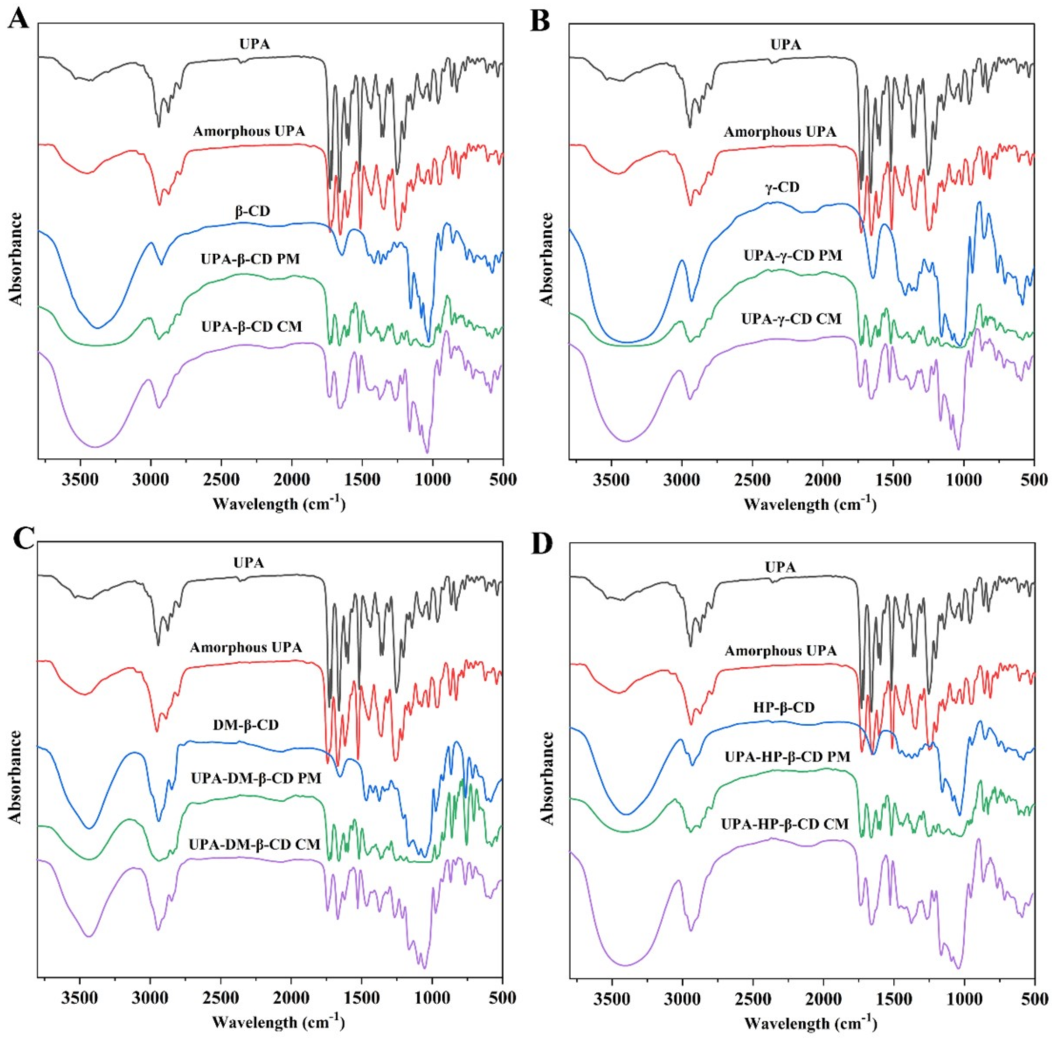

Fig. S4 FT-IR spectra of UPA-β-CD (A), UPA-γ-CD (B), UPA-DM-β-CD (C), and UPA-HP-β-CD (D). Each picture from top to bottom: crystalline UPA, amorphous UPA, CD, UPA–CD PM, and UPA–CD CM.

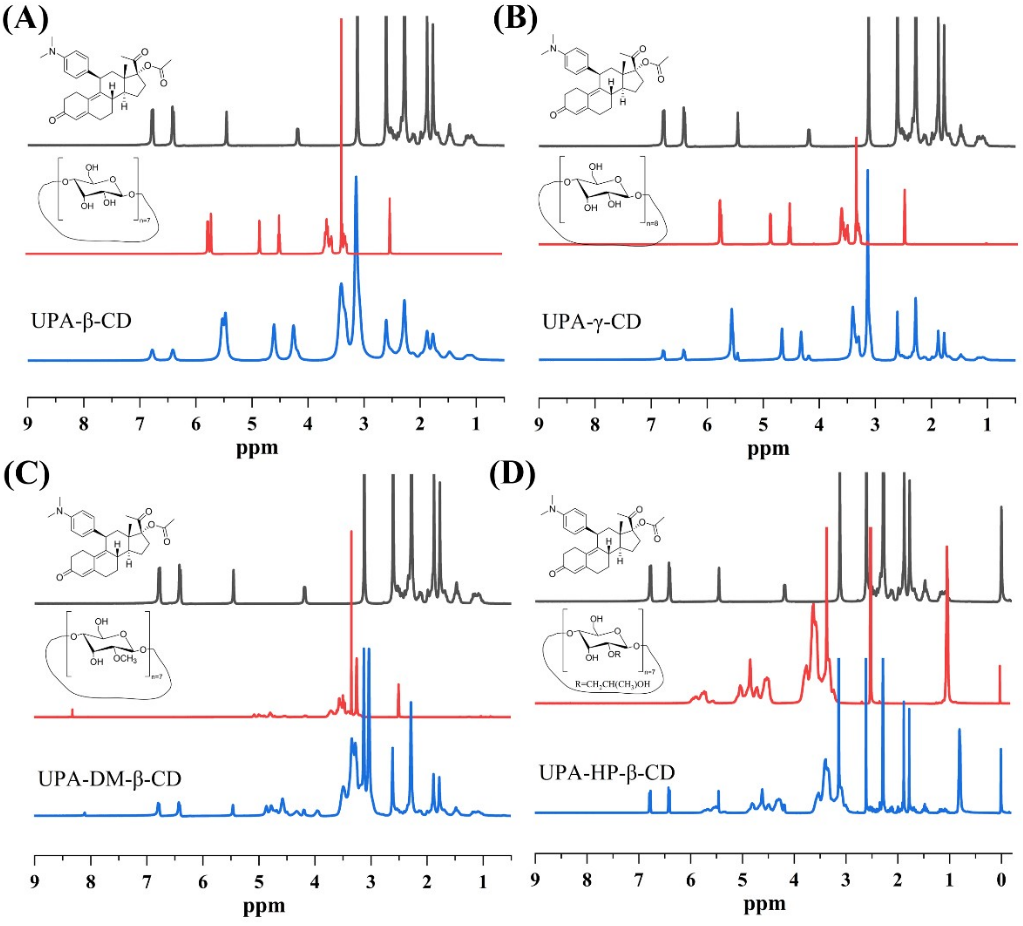

Fig. S5 ¹H NMR spectra of UPA, CDs, and UPA-β-CD (A), UPA-γ-CD (B), UPA-DM-β-CD (C), and UPA-HP-β-CD (D).

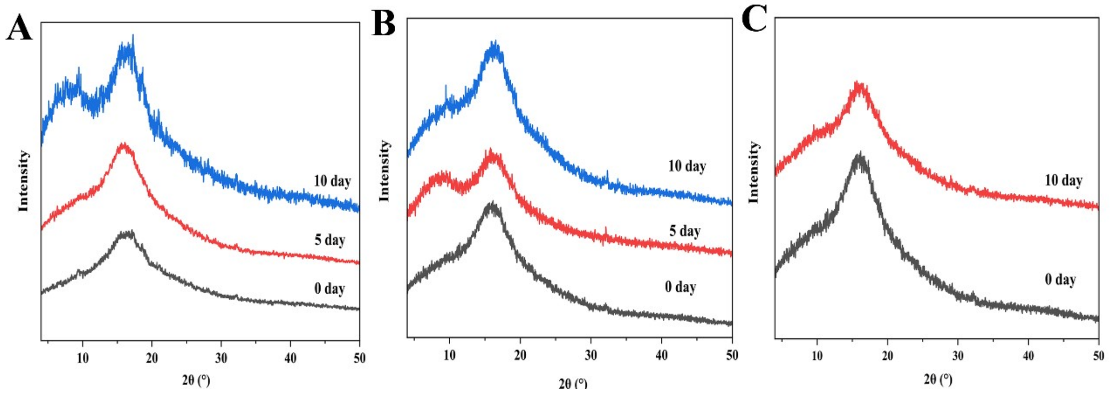

Fig. S6 Stability of amorphous UPA under high temperature (A), high humidity (B), and light (C) conditions.

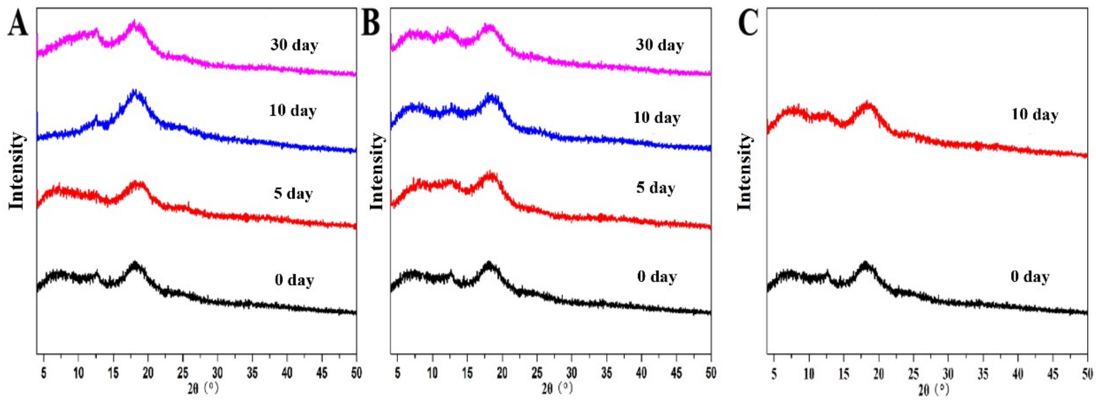

Fig. S7 Stability of UPA-β-CD amorphous at high temperature (A), high humidity (B), and light (C).

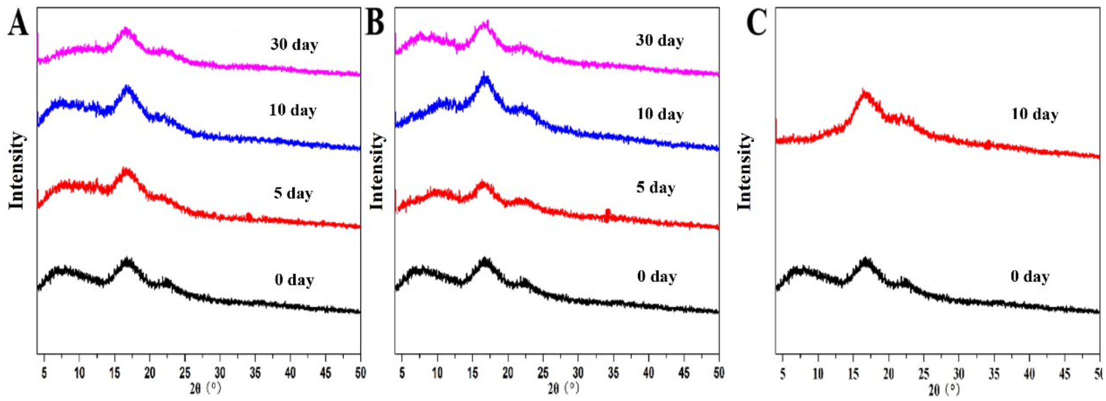

Fig. S8 Stability of UPA-γ-CD amorphous at high temperature (A), high humidity (B), and light (C).

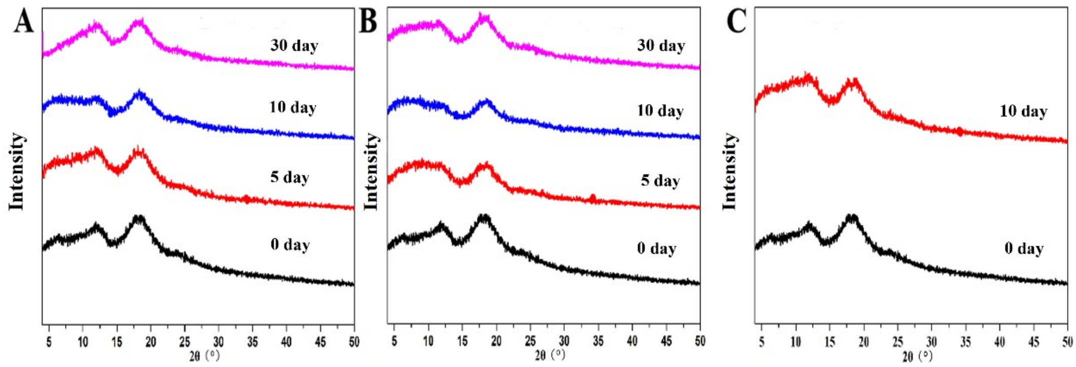

Fig. S9 Stability of UPA-DM-β-CD amorphous at high temperature (A), high humidity (B), and light (C).

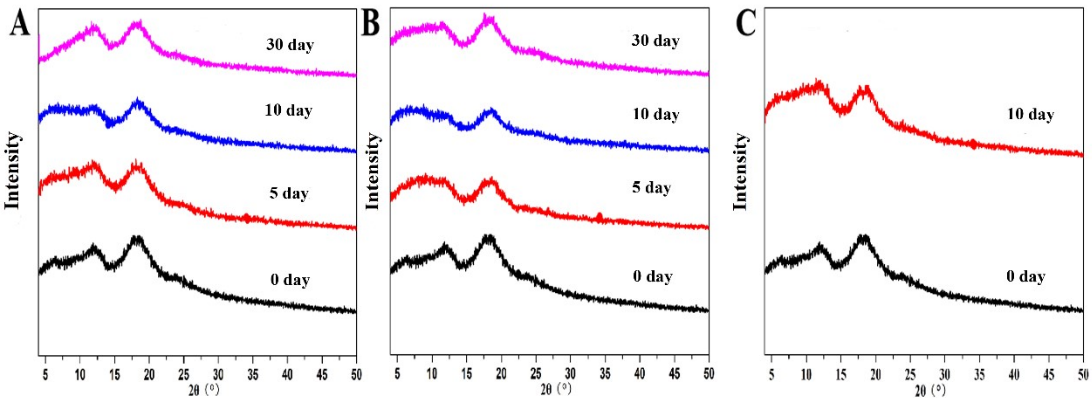

Fig. S10 Stability of UPA-HP-β-CD amorphous at high temperature (A), high humidity (B), and light (C)
